# FGFR1 inhibition by carvacrol: A novel strategy for oral squamous cell carcinoma therapy

**DOI:** 10.1016/j.gendis.2024.101479

**Published:** 2024-12-04

**Authors:** Shuzhen Xiang, Hongyan Zhang, Qian Wang, Jiajia Fan, Shiheng Jia, Lan Zhang, Wei Ma, Minda Liu, Yanshu Li, Wei Dai

**Affiliations:** a Department of Oral Maxillofacial-Head and Neck Surgery, School of Stomatology, China Medical University, Oral Diseases Laboratory of Liaoning, Shenyang, Liaoning 110000, China; b Department of Cell Biology, Key Laboratory of Cell Biology, Ministry of Public Health, and Key Laboratory of Medical Cell Biology, Ministry of Education, China Medical University, Shenyang, Liaoning 110122, China; c Department of Surgical Oncology and General Surgery, The First Hospital of China Medical University, Shenyang, Liaoning 110001, China; d Key Laboratory of Molecular Pathology and Epidemiology of Gastric Cancer in the Universities of Liaoning Province, Shenyang, Liaoning 110001, China; e School of Stomatology, China Medical University, Shenyang, Liaoning 110122, China

Poor prognosis is associated with oral squamous cell carcinoma (OSCC), an aggressive form of malignant tumor.^1^ This study aimed to investigate the pharmacological effects of carvacrol on OSCC by targeting the tumor-associated antigen FGFR1. As a key survival biomarker in OSCC, FGFR1 plays a crucial role in malignant transformation. Carvacrol, a specific FGFR1 inhibitor, induces its degradation via the ubiquitin-proteasome pathway.

Carvacrol (CV), a natural monoterpenoid phenolic compound (Fig. 1A) with reported anti-cancer properties in OSCC cells, suppresses malignant proliferation.^2^ Colony formation (Fig. 1B; Fig. S1A) and CCK8 assays (Fig. S1B) assessed colony formation and viability (IC50) in Cal27, HSC2, and HSC4 cells after carvacrol treatment. Carvacrol significantly reduced cell growth and colony formation. Scratch (Fig. 1C; Fig. S1C) and transwell assays (Fig. 1D; Fig. S1D) showed that 80 μM carvacrol inhibited OSCC migration and invasion, highlighting its role in suppressing OSCC proliferation and metastasis.

**Figure 1:**
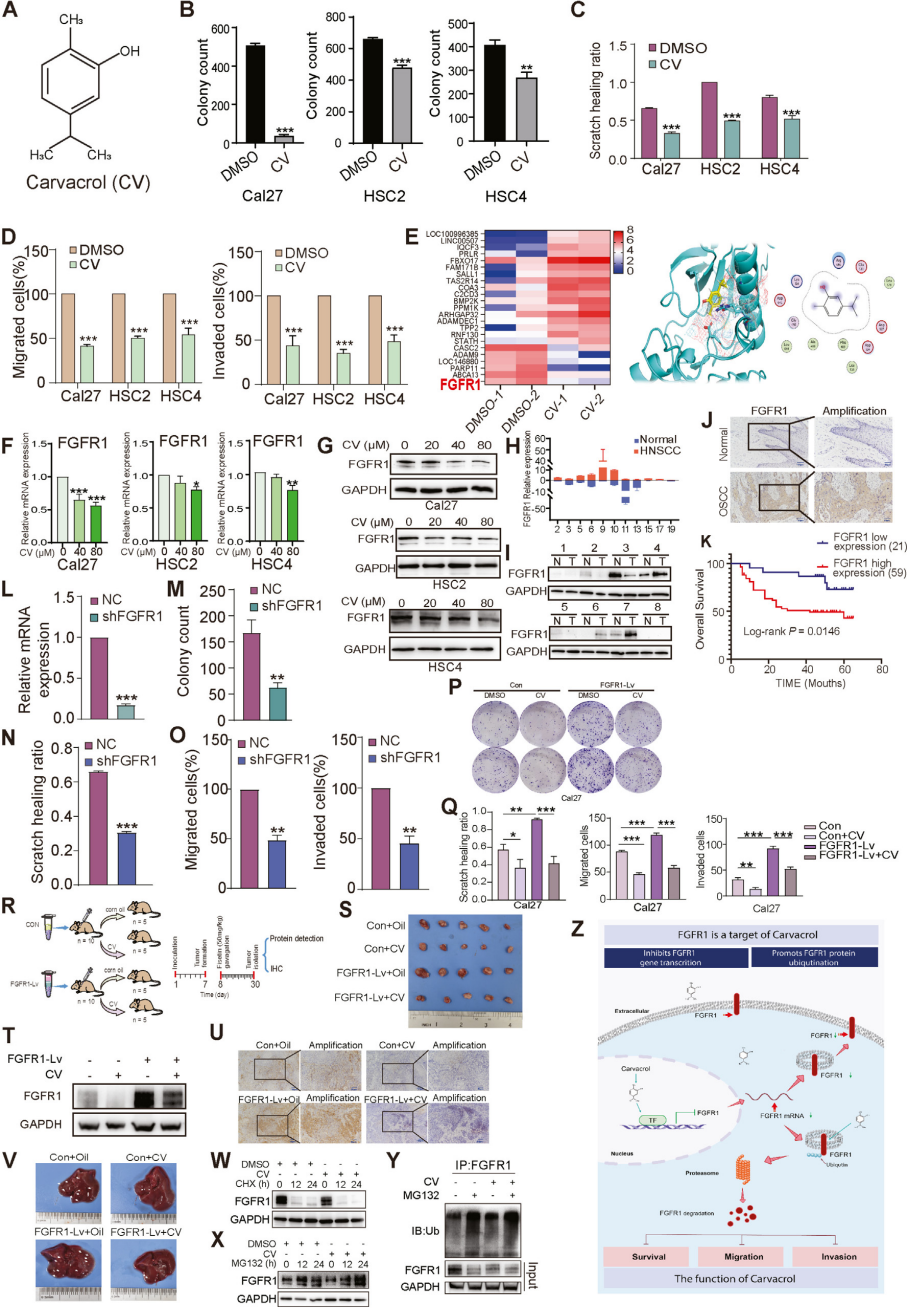
The biological effects and mechanisms of carvacrol (CV) for oral squamous cell carcinoma (OSCC) therapy by the inhibition of FGFR1. **(A)** Chemical structure of CV. **(B)** Colony-formation assay of OSCC cancer cells treated with DMSO or CV. **(C)** The scratch assay detected the healing at 24 h (HSC2 and HSC4) or 48 h (Cal27). **(D)** Migration and invasion of OSCC cells determined by transwell assays. **(E)** Heatmaps of partially differentially expressed genes after whole genome sequencing. Molecular docking analysis of the three-dimensional spatial conformation of CV and FGFR1. **(F)** Real-time PCR analysis of FGFR1 gene expression after different doses of CV treatment, with GAPDH as the reference. **(G)** Western blot analysis of FGFR1 protein expression after different doses of CV treatment. The bar charts show statistical analysis of the ratio of FGFR1 to GAPDH protein grayscale values. **(H)** Real-time PCR detection of FGFR1 gene expression in normal and tumor tissues adjacent to cancer in 11 pairs of OSCC patients. **(I)** Western blot analysis of FGFR1 protein expression in adjacent normal tissues (N) and tumor tissues (T) of 8 pairs of OSCC patients. **(J)** Immunohistochemical detection of FGFR1 expression in normal and OSCC tissues adjacent to cancer. **(K)** Overall survival analysis of 80 patients based on FGFR1 expression detected by immunohistochemistry in OSCC. **(L)** Real-time PCR analysis of mRNA expression in Cal27 cells with stable FGFR1 knockdown. **(M)** Clone formation assay assessed the proliferation of FGFR1-silenced OSCC cells. **(*n*)** Scratch assay evaluated the migration of FGFR1-silenced OSCC cells. **(O)** Transwell assay measured the migration and invasion of FGFR1-silenced OSCC cells. **(P, Q)** Clone formation, scratch, and transwell assays were performed to assess the function of FGFR1 and the effect of CV treatment. **(R)** A diagram of the timeline of tumor formation in BALB/c nude mice. **(S)** Xenograft tumor experiments were conducted using Cal27 cells overexpressing FGFR1 in nude mice, with CV administered orally after tumor formation to observe tumor growth volume. **(T)** Western blot analysis of FGFR1 protein expression in tumor tissues from each treatment group. **(U)** Immunohistochemical analysis of FGFR1 expression in tumor tissues from each treatment group. **(V)** Representative images of liver tissues from each treatment group. **(W)** Western blot analysis of FGFR1 degradation in OSCC cells treated with DMSO or CV, in combination with cycloheximide (CHX). **(X)** Western blot analysis of FGFR1 levels in OSCC cells treated with DMSO or CV, in combination with the proteasome inhibitor MG132. **(Y)** Western blot detection of ubiquitinated FGFR1 in OSCC cells treated with CV and MG132. **(Z)** Proposed model of CV regulation of FGFR1 gene and protein expression.

To further investigate the molecular effects of carvacrol, transcriptomic analysis was performed after DMSO (control) or 80 μM carvacrol treatment using nanopore sequencing. FGFR1, a transmembrane receptor tyrosine kinase, is critical in various cellular processes.^3^ The three-dimensional structural analysis and molecular docking showed that carvacrol occupied the FGFR1 interaction pocket, engaging multiple amino acids (Fig. 1E). Real-time PCR confirmed carvacrol down-regulated FGFR1 expression at 40 μM and 80 μM in Cal27, HSC2, and HSC4 cells (Fig. 1F), while western blotting revealed a concentration-dependent FGFR1 protein reduction (Fig. 1G). These results suggest FGFR1 as a target of carvacrol, supporting its therapeutic potential in OSCC.

FGFR1 consists of an extracellular ligand-binding domain, a transmembrane helix, and an intracellular tyrosine kinase domain (Fig. S2A).^4^ We analyzed FGFR1 gene (Fig. S2B) and protein (Fig. S2C) expression in HaCat cells and OSCC lines (Cal27, HSC2, HSC4, SCC9), and mRNA in 11 OSCC/adjacent normal tissue pairs (Fig. 1H), showing higher expression in 63.64% of tumors. Protein analysis in 8 tissue pairs (Fig. 1I) and immunohistochemistry of 80 OSCC samples (Fig. 1J) confirmed elevated FGFR1, mainly in membranes and cytoplasm. Survival analysis (Fig. 1K) linked high FGFR1 levels to poor prognosis, identifying it as a therapeutic target in OSCC.

We generated stable FGFR1 knockdown Cal27 cells using lentivirus (shFGFR1) with NC as control. Western blot and PCR confirmed FGFR1 silencing (Fig. 1L; Fig. S3A). Colony formation assays showed reduced colonies (Fig. 1M; Fig. S3B), while scratch and transwell assays showed impaired migration and invasion in shFGFR1 cells (Fig. 1N, O; Fig. S3C, D). These findings emphasize the key role of FGFR1 in OSCC proliferation, migration, and invasion, highlighting its potential as a therapeutic target.

To validate the inhibitory effect of carvacrol on FGFR1-driven malignancy, we engineered FGFR1-lentivirus particles to infect Cal27 cells. FGFR1 expression was confirmed by Western blot and PCR (Fig. S4A, B). Colony formation assays showed that carvacrol (80 μM) significantly inhibited FGFR1-induced proliferation (Fig. 1P), while scratch and transwell assays confirmed that carvacrol suppressed FGFR1-mediated migration and invasion (Fig. 1Q; Figs. S4C and D). These results confirm the efficacy of carvacrol in inhibiting FGFR1-induced OSCC malignancy.

We examined the effect of carvacrol on tumor growth *in vivo* using an OSCC xenograft model. Nude mice were injected with Cal27 cells infected with Con or FGFR1-Lv lentivirus and treated with vehicle (corn oil) or carvacrol (100 mg/kg) (Fig. 1R). FGFR1 overexpression significantly increased tumor growth, while carvacrol inhibited FGFR1-driven tumor proliferation (Fig. 1S–U). Additionally, FGFR1 overexpression promoted liver metastasis, which was significantly reduced by carvacrol (Fig. 1V). These results confirmed that carvacrol inhibited FGFR1-mediated OSCC proliferation and metastasis.

To confirm that carvacrol induced FGFR1 degradation via the ubiquitin-proteasome pathway, a CHX chase assay was performed. Carvacrol-treated OSCC cells showed accelerated FGFR1 degradation (Fig. 1W). MG132 treatment increased FGFR1 levels (Fig. 1X), confirming proteasome involvement. Immunoprecipitation revealed enhanced FGFR1 ubiquitination in carvacrol-treated cells, especially with MG132 (Fig. 1Y). A schematic (Fig. 1Z) illustrates how carvacrol regulates FGFR1 via transcriptional control and ubiquitin-proteasome degradation, suppressing cancer cell proliferation, migration, and invasion.

## Ethics declaration

All the samples were obtained from the Hospital of Stomatology of China Medical University. All animal experiments were approved by the Animal Care Committee of China Medical College (approval number: KT20240844).

## Funding

This work was supported by the 10.13039/501100001809National Natural Science Foundation of China (No. 81902701 to Y. Li; 82103649 to H. Zhang), the 10.13039/501100005047Natural Science Foundation of Liaoning Province, China (No. 20180530037, 2023JH2/20200036 to Y. Li; 2022-MS-183 to W. Dai).

## CRediT authorship contribution statement

**Shuzhen Xiang:** Writing – original draft, Data curation, Conceptualization. **Hongyan Zhang:** Methodology, Investigation, Funding acquisition. **Qian Wang:** Software, Methodology, Conceptualization. **Jiajia Fan:** Investigation. **Shiheng Jia:** Methodology, Formal analysis. **Lan Zhang:** Methodology. **Wei Ma:** Software. **Minda Liu:** Writing – review & editing. **Yanshu Li:** Writing – review & editing, Writing – original draft, Supervision, Methodology, Funding acquisition, Data curation, Conceptualization. **Wei Dai:** Writing – review & editing, Writing – original draft, Methodology, Investigation, Funding acquisition, Conceptualization.

## Data availability

The authors confirm that the data supporting the findings of this study is available within the article and its supplementary materials.

## Conflict of interests

The authors declared no conflict of interests.
