# Supplementary material for: FGFR1 inhibition by carvacrol: A novel strategy for oral squamous cell carcinoma therapy
Source: Genes Dis. 2024 Dec 4;12(5):101479. doi: 10.1016/j.gendis.2024.101479 (PMC12142515; doi:10.1016/j.gendis.2024.101479)
Supplement: Multimedia component 1 [file mmc1.docx]

**Supplementary Material and Methods**

**Cell culture and cultures**

Human OSCC cell lines Tca8113 (RPMI-1640 medium) and Cal27 (DMEM medium) were obtained from Shanghai Genechem. HSC2 (MEM medium), HSC4 (DMEM medium), and SCC9 (DMEM/F12 medium) were purchased from Jiangsu KeyGEN BioTECH. The human OSCC cell lines were cultured in the indicated media with 10% FBS (VivaCell).

**Chemical compounds**

Carvacrol (CV) was provided by Sigma-Aldrich (St Louis, MO, USA) and dissolved in dimethyl sulphoxide (DMSO). Cycloheximide (CHX, HY-12320) and the proteasome inhibitor MG132 (HY-13259) were purchased from MedChemExpress.

**Production of lentivirus for FGFR1 overexpression and knockdown**

Human FGFR1 lentivirus (FGFR1-Lv), negative control (CON), shRNA lentivirus against FGFR1 (shFGFR1), and scrambled shRNA lentivirus (NC) were purchased from GENECHEM (Shanghai, China). Stable cell lines were generated by infecting cells with lentiviruses.

**OSCC patient samples and Immunohistochemical**

We collected 80 OSCC tissues from China Medical University Stomatological Hospital with complete clinicopathologic characteristics and follow-up data were enrolled in the study. This study was approved by the Medical Ethics Committee of the Affiliated Stomatological Hospital of China Medical University (No. 2023027). All participants in this study provided informed consent before undergoing the procedure. First, the tissue sections were deparaffinized and rehydrated. Endogenous peroxidase activity was quenched with H_2_O_2_. After washing with TBST, the slides were blocked with goat serum, followed by incubation with the primary antibody and the secondary antibody in a humidified chamber. The target protein was visualized using a DAB substrate, and hematoxylin was used for counterstaining.

**Western blotting analysis**

The primary antibodies used for western blotting were as follows: FGFR1 (#9740), E-cadherin (#14472S), and N-cadherin (#14215S) from Cell Signaling Technology (Beverly, MA); GAPDH (60004-1-Ig) and FGFR1 (60325-1-Ig) from proteintech biotechnology (Wuhan, China). The intensity of each protein band was analyzed using ImageJ software, normalized to GAPDH or its non-phosphorylated form, and expressed as a percentage of vehicle-treated controls to quantify protein expression levels.

**Quantitative real-time PCR**

Quantitative real-time PCR was performed on a StepOne Real-Time PCR System (Applied Biosystems) using SYBR Premix Ex Taq (TaKaRa). The specific primer sequences were as follows: 5′- GCCCAGACAACCTGCCTTAT -3′ (forward) and 5′-CACGTATACTCCCCTGCGTC-3′ (reverse) for human FGFR1; 5′-CAGGAGGCATTGCTGATGAT-3′ (forward) and 5′-GAAGGCTGGGGCTCATTT-3′ (reverse) for human GAPDH.

**Colony formation assay**

400 cells were seeded and cultured for 14 days. Colonies were then fixed, stained, and counted.

**CCK8 assay and IC50 calculation**

5 × 10^3^ cells in 100 μL of medium were seeded and switched to medium containing different concentrations of CV on the second day. After 24 h of cultivation, the medium was replaced with mixed medium containing 10% CCK8. Absorbance was measured at 450 nm using a spectrophotometer, and the IC50 value was calculated using GraphPad Prism software. For the proliferation assay, 1000 cells per well were seeded and were collected at the indicated time points, and the OD was measured at 450 nm.

**Scratch assay**

Cells in the 12-well plates formed a confluent monolayer, a scratch was made using 200 μL pipette tips. The cells were then cultured in medium containing 1% FBS and various reagents.

**Transwell migration and invasion assay**

1 × 10^5^ cells seeded in upper chamber with 100 μl medium containing 1% FBS. The lower chamber was filled with 600 μL of medium containing 20% FBS.

***In vivo* tumor growth assay**

CAL27 cells (2 × 10^6^ cells in 50 μL PBS) stably transfected with an FGFR1 expression vector, or an empty vector were injected subcutaneously into the right flanks of 4 to 6-week-old athymic nude mice (n = 5). When the tumor volume reached 100 mm³, carvacrol (100 mg/kg) or an equal volume of vehicle was administered by oral gavage daily for 3 weeks. The excised tissues were fixed in 4% paraformaldehyde (PFA) and embedded in paraffin for subsequent experiments. All experimental procedures were approved by the Animal Ethics Committee of China Medical University (KT2023074).

**Statistical analysis**

All statistical calculations and curve fitting were conducted using GraphPad Prism 9.0.2 (San Diego, CA). Results are presented as mean ± SD. Statistical significance between groups was analyzed using the paired Student’s *t*-test or one-way ANOVA, as appropriate. A *P*-value of < 0.05 was considered statistically significant.
