# Supplementary material for: FGFR1 inhibition by carvacrol: A novel strategy for oral squamous cell carcinoma therapy
Source: Genes Dis. 2024 Dec 4;12(5):101479. doi: 10.1016/j.gendis.2024.101479 (PMC12142515; doi:10.1016/j.gendis.2024.101479)
Supplement: Multimedia component 2 [file mmc2.docx]

**Supplementary figures and tables**


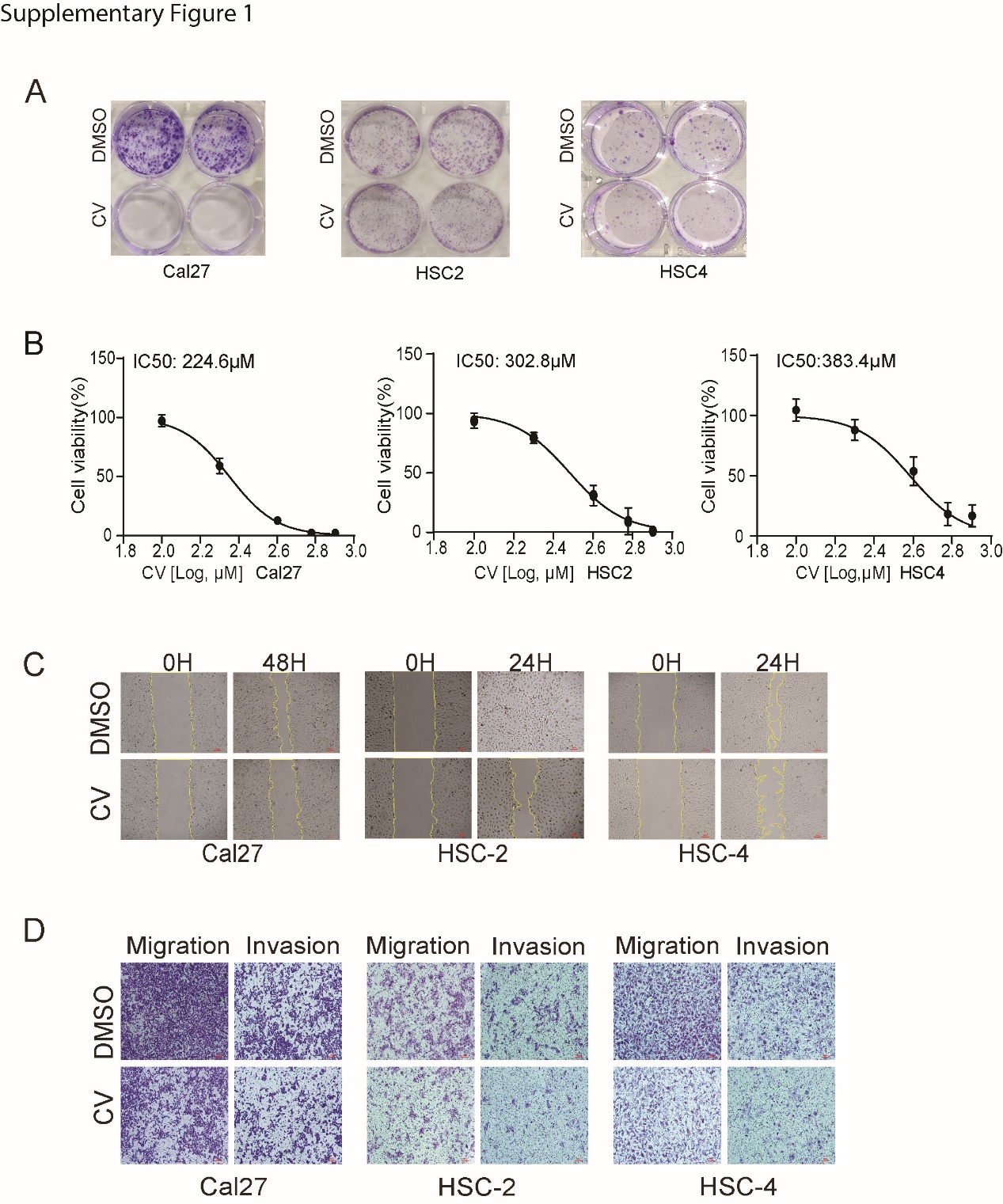


**Figure S1.** Carvacrol displays anti-OSCC Activity *in vitro.* **(A)** Colony formation assay was performed to assess the long-term effects of Carvacrol (CV) on the proliferation of oral squamous cell carcinoma (OSCC) cell lines Cal27, HSC2, and HSC4. Cells were treated with DMSO (control) or CV for a defined period, followed by crystal violet staining to visualize colonies. **(B)** The IC_50_ values of CV was measured by CCK-8 assay in OSCC cells. **(C)** The scratch assay was used to detect the healing of 24 hours or 48 hours, respectively. **(D)** Transwell migration and invasion assays were performed to assess the effects of CV on the migratory and invasive capacities of OSCC cell lines Cal27, HSC2, and HSC4.


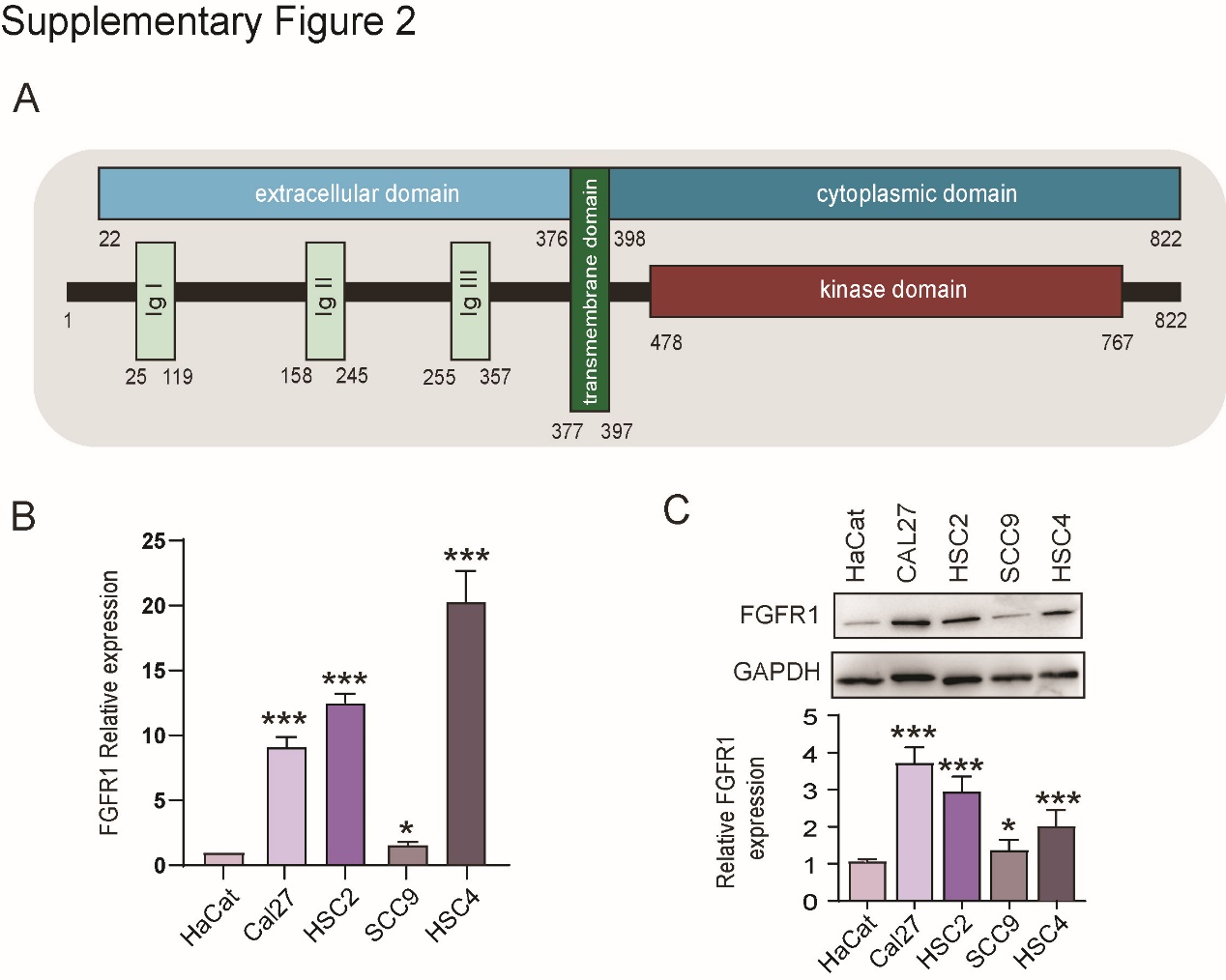


**Figure S2.** FGFR1 structure and expression in OSCC cell lines. **(A)** Schematic representation of the structural domains of the FGFR1 protein. **(B)** Quantitative RT-PCR analysis of FGFR1 mRNA expression levels in HaCat (normal keratinocytes) and OSCC cell lines (Cal27, HSC2, SCC9, HSC4). **(C)** Western blot analysis of FGFR1 protein levels in HaCat and OSCC cell lines. GAPDH serves as a loading control. (**p* < 0.05, ****p* < 0.001).


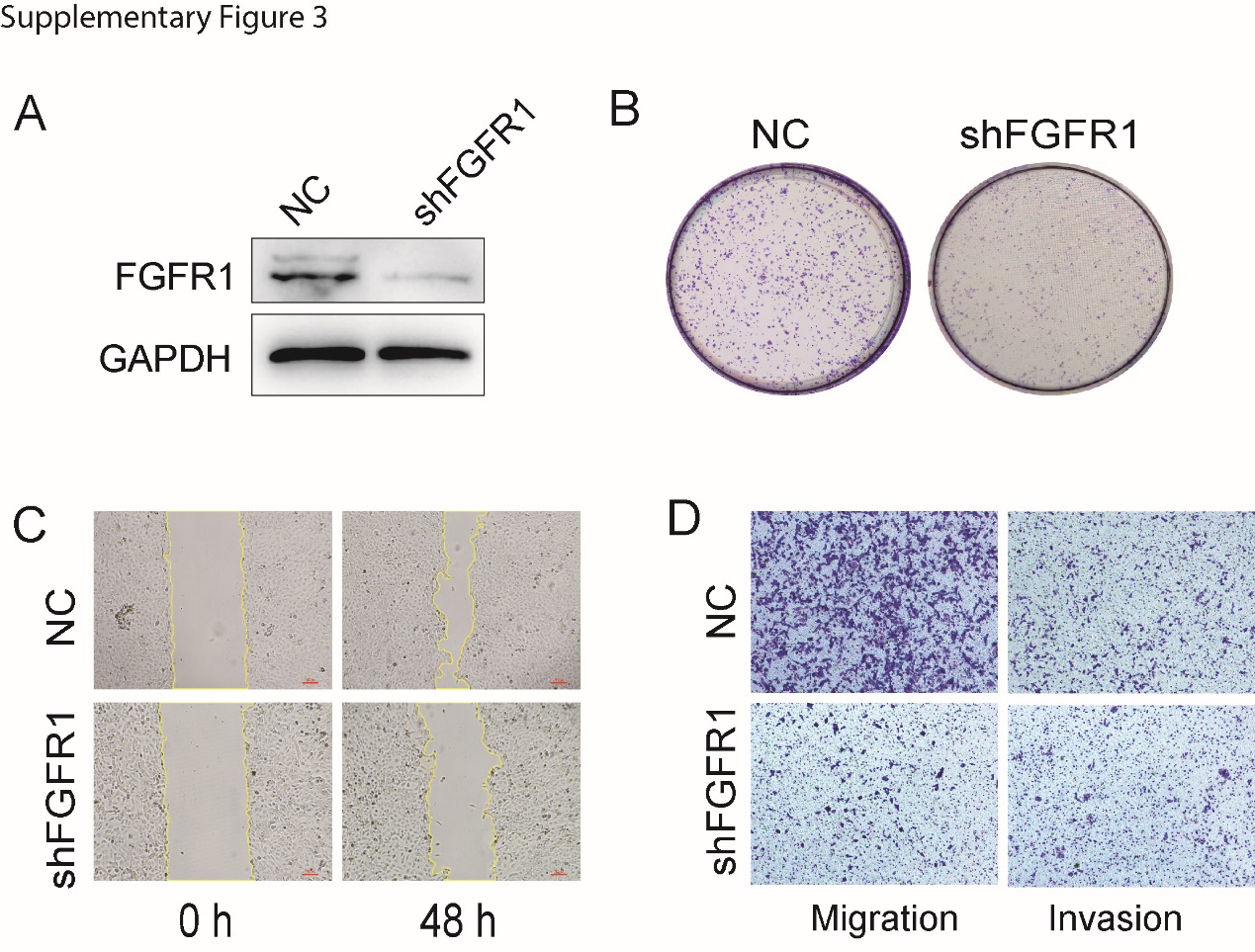


**Figure S3**. Silencing of FGFR1 suppresses proliferation, migration, and invasion in Cal27 cells. **(A)** Western blot analysis of FGFR1 protein expression in cells transfected with non-targeting control (NC) or shRNA targeting FGFR1 (shFGFR1). GAPDH is used as a loading control. **(B)** Colony formation assay of OSCC cells transfected with NC or shFGFR1. **(C)** Wound healing assay showing the migration of cells transfected with NC or shFGFR1 at 0 and 48 hours. **(D)** Transwell migration and invasion assays of OSCC cells transfected with NC or shFGFR1.


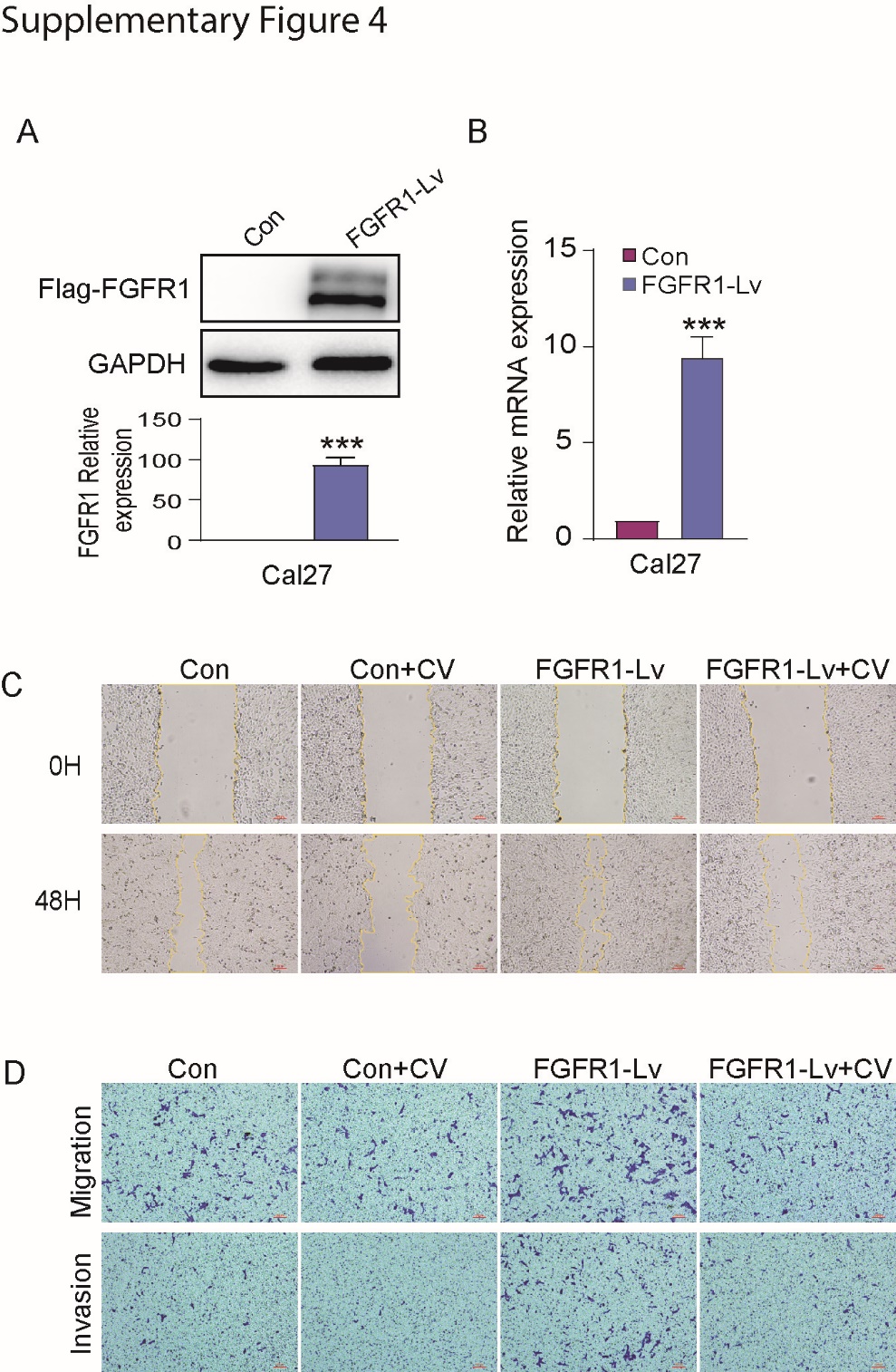


**Figure S4**. Overexpression of FGFR1 in cal27 cells enhances migration and invasion and reduces sensitivity to carvacrol. **(A)** Western blot analysis of FGFR1 protein expression in Cal27 cells transfected with control (Con) or FGFR1 overexpression lentivirus (FGFR1-Lv). GAPDH is used as a loading control. **(B)** Quantitative RT-PCR analysis of FGFR1 mRNA expression in Cal27 cells transfected with control (Con) or FGFR1 overexpression lentivirus (FGFR1-Lv). **(C)** Wound healing assay to assess the migration ability of Cal27 cells transfected with control (Con), treated with Carvacrol (Con+CV), transfected with FGFR1 overexpression lentivirus (FGFR1-Lv), and combined treatment of FGFR1 overexpression and Carvacrol (FGFR1-Lv+CV). Wound closure was observed at 0 hours and 48 hours after scratching the cell monolayer. **(D)** Transwell migration and invasion assays showing the effects of FGFR1 overexpression and Carvacrol treatment on the migratory and invasive capacities of Cal27 cells.
